# Arcuate fracture of the fibular head: two-tunnel surgical technique

**DOI:** 10.1051/sicotj/2026052

**Published:** 2026-10-07

**Authors:** Bujar Shabani, Dijon Musliu, Dafina Bytyqi, Rosa Ballis

**Affiliations:** 1 Clinic of Orthopedics, University Clinical Center of Kosovo Prishtina Kosovo; 2 Faculty of Medicine, University of Prishtina “Hasan Prishtina” Prishtina Kosovo; 3 Ortopedia e Traumatologia, Ospedale San Martino Belluno Italy

**Keywords:** Arcuate fracture, Surgical technique, PLC, Fibular head

## Abstract

Avulsion fractures of the fibular head, commonly manifesting as the “arcuate sign” on radiographs, are indicative of posterolateral corner (PLC) injuries of the knee and, if inadequately managed, may lead to chronic instability and degenerative joint changes. This technical note describes a novel surgical technique for the fixation of arcuate fractures following failed anchor repair. The procedure involves dual tunnel formation in the fibula and strategic FiberTape passage to achieve anatomical reduction and stable fixation of the avulsed fragment. Key advantages of this approach include a low-profile construct, reproducibility, minimized soft tissue irritation, and lower initial implant-related cost. The technique also allows for restoration of PLC integrity while minimizing the risk for failure. Challenges such as potential unequal suture tension are acknowledged but may be mitigated through meticulous surgical execution. This method represents a reliable and effective alternative for managing unstable arcuate fractures, particularly in cases involving complex PLC injury patterns. Further studies are recommended to validate long-term outcomes and optimize treatment strategies.

## Introduction

An avulsion fracture of the fibular head is a well-recognized radiographic marker of posterolateral corner (PLC) instability of the knee. This injury, commonly referred to as the “arcuate sign,” represents an avulsion involving the arcuate complex, which consists of the fabellofibular ligament, popliteofibular ligament, and lateral collateral ligament (LCL) [1, 2]. On plain radiographs, the arcuate sign appears as a fracture of the proximal fibula and is highly suggestive of underlying PLC injury [3, 4].

The injury mechanism typically involves a direct blow to the anteromedial aspect of the knee with the tibia in external rotation, or a hyperextension injury combined with internal tibial rotation [5]. Additionally, these fractures may occur following direct trauma producing excessive varus stress with internal rotation, or indirect mechanisms that generate similar force vectors across the posterolateral structures [6].

If left untreated or inadequately managed, PLC injuries can lead to chronic knee instability, progressive degenerative changes, persistent varus laxity, and abnormal external tibial rotation [7]. Thus, the indications for this surgical treatment include acute arcuate fracture as well as failed primary fixation as revision (Figure 1)

**Figure 1:**
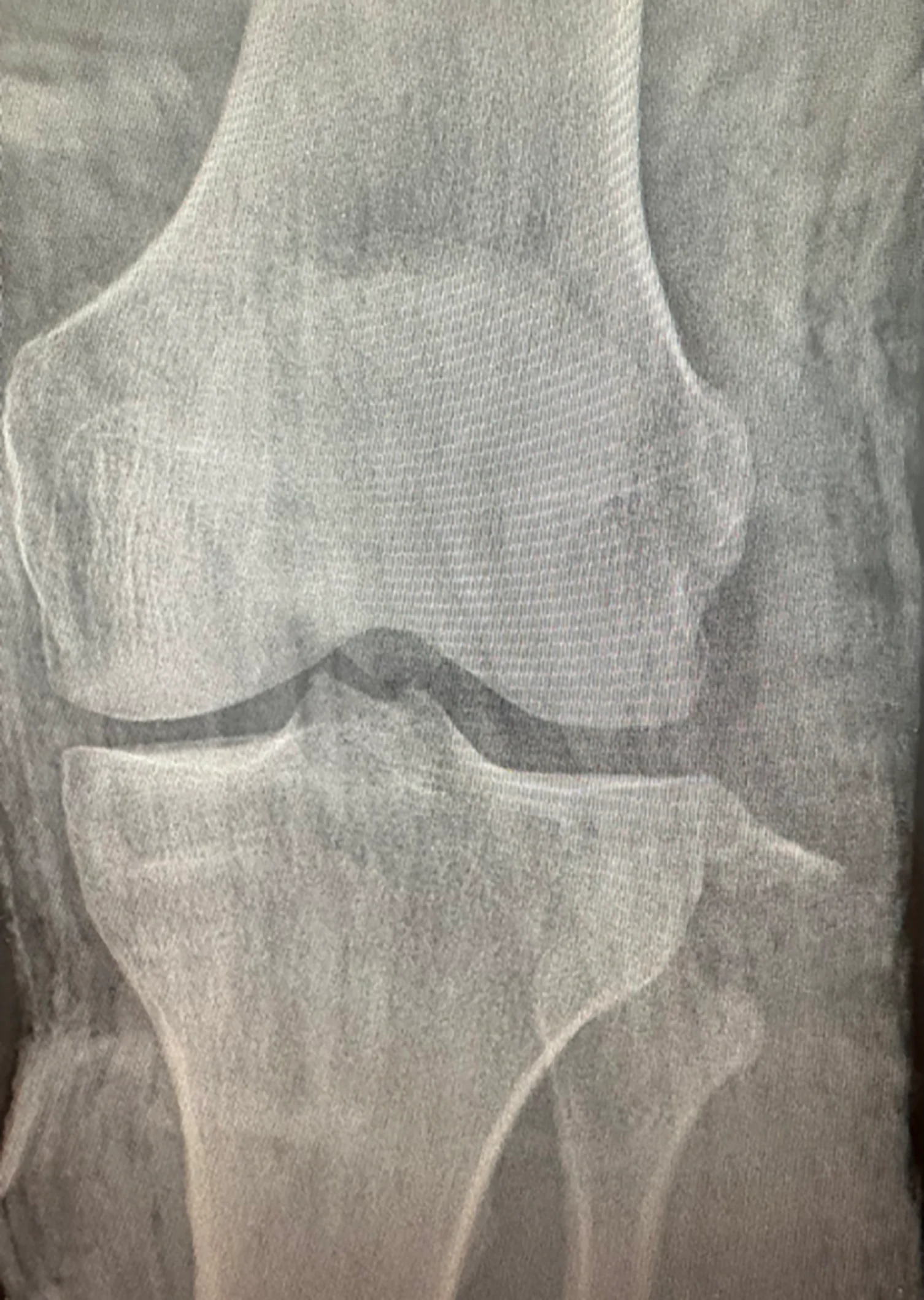
(A, B, C). Schematic illustration of the surgical technique demonstrating tunnel placement and suture configuration. Blue – first tunnel; Red – second tunnel.

The purpose of this technical note is to describe a novel surgical technique for the fixation of fibular head avulsion fractures after failed anchor fixation, aiming to restore PLC integrity and achieve stable fracture healing.

### Surgical technique

#### Patient positioning

The patient is placed in the supine position. The operative knee is flexed to 90° and allowed to hang freely over the edge of the operating table. The contralateral limb is positioned in a padded lithotomy leg holder to protect bony prominences and neurovascular structures.

#### Surgical approach

A longitudinal incision is made over the lateral aspect of the knee, extending from the lateral femoral epicondyle to the proximal fibula. Dissection proceeds through the subcutaneous tissues to the upper part of the proximal fibula fragment, where the posterolateral corner (PLC) is attached. The biceps femoris tendon (BFT) and the lateral collateral ligament (LCL), both of which insert onto the arcuate fragment, are identified. The tension generated by these structures often contributes to proximal migration of the fragment and increased displacement. The common peroneal nerve is identified posterior to the biceps femoris tendon and is meticulously protected throughout the procedure. The fracture site is then exposed, including the arcuate fragment.

#### Tunnel formation and fixation technique

Two bone tunnels are created in the distal portion of the fibular fracture bed 1.5 cm distally from the fracture site (Figure 2, Schematic drawing):

**Figure 2:**
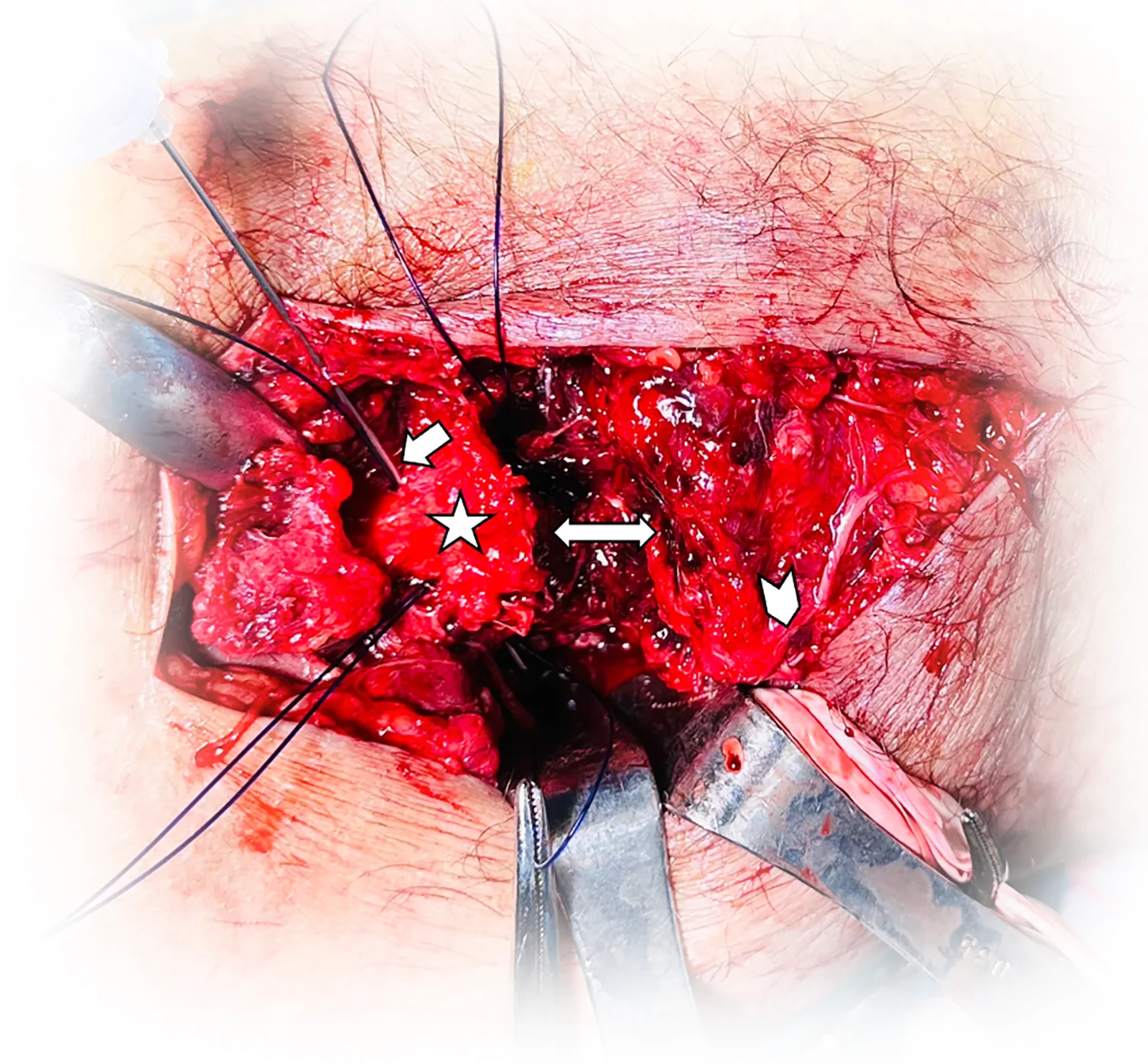
Intraoperative image demonstrating identification and protection of the common peroneal nerve (arrowhead), exposure of the fracture site (double-headed arrow), the arcuate fragment on the right side, distal fibula (*), and passage of the suture through the previously created tunnel (arrow).

1. *Tunnel 1* is drilled, from the anteromedial cortex to the posterolateral cortex of the fibula.
2. *Tunnel 2* is drilled from the anterolateral cortex into the fibular medullary canal.

A nonabsorbable suture tape (FiberTape, Arthrex, Naples, FL, USA) is passed through Tunnel 1, traversing the fibula completely. A second suture tape (FiberTape, Arthrex, Naples, FL, USA) is introduced through Tunnel 2 into the medullary canal. The intramedullary end of the second suture tape (Arthrex, Naples, FL, USA) is then passed through the middle avulsed fragment, using a threadless needle, facilitating anatomic reduction of the fracture (Figures 3 and 4)

**Figure 3:**
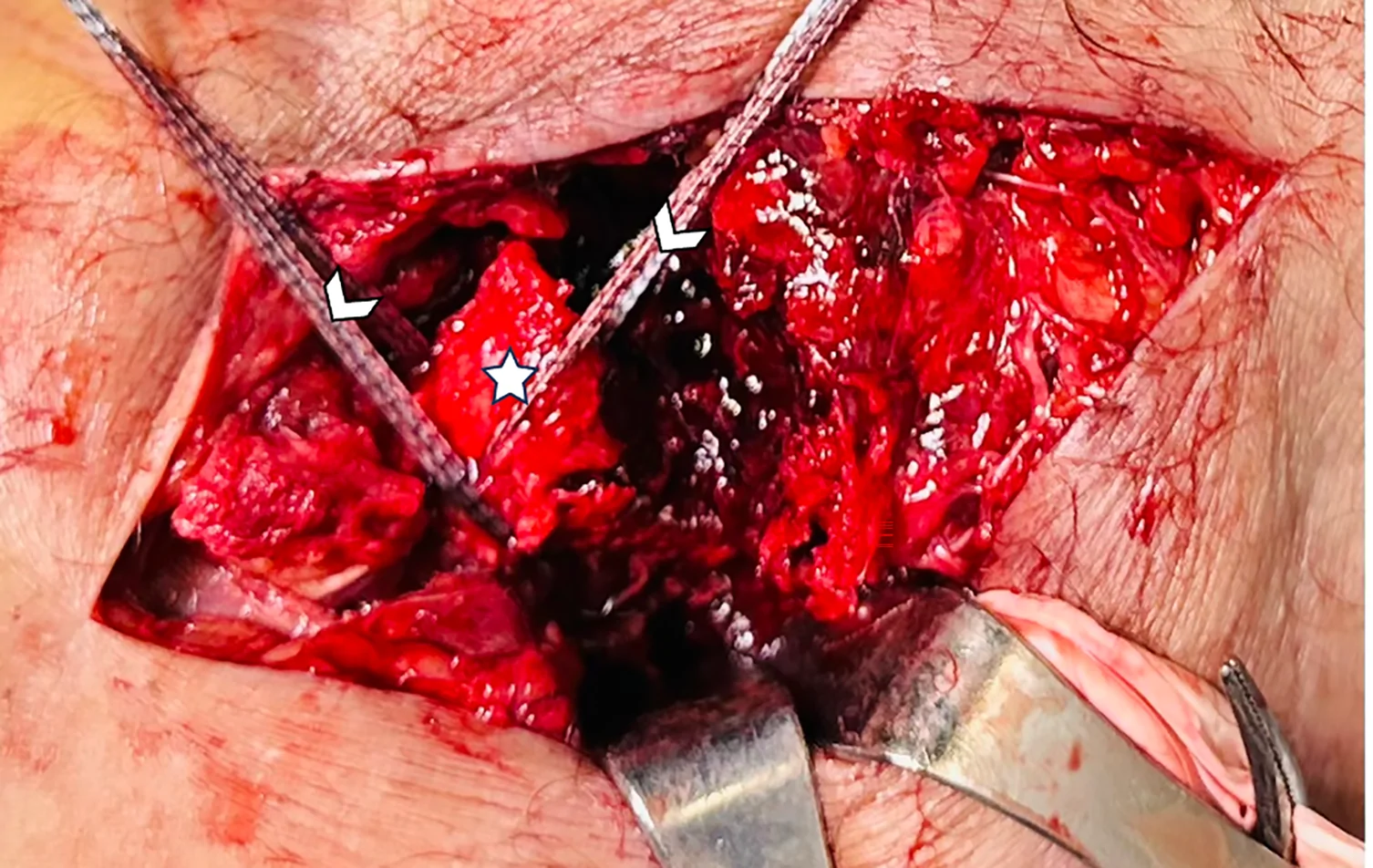
Intraoperative image demonstrating passage of the Fiber Tape (arrowheads, Arthrex) through both tunnels before advancement to the proximal (arcuate) fragment. The asterisk indicates the distal fibula.

**Figure 4:**
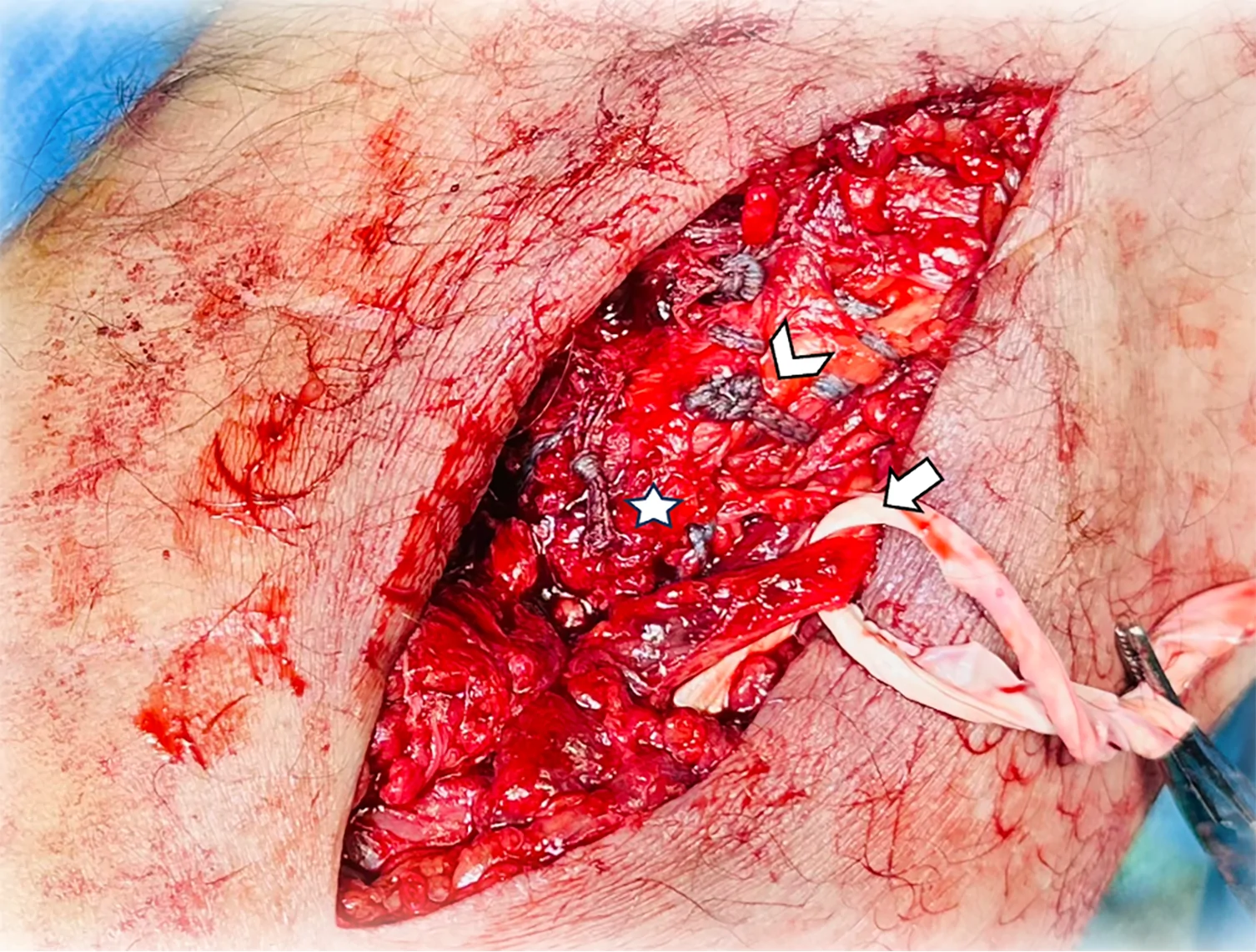
Completed fixation with the reduced distal fibula (*), protected common peroneal nerve (arrow), and FiberTape securing both fracture fragments (arrowhead).

Both free ends of the second FiberTape (Arthrex, Naples, FL, USA) are subsequently passed through the lateral collateral ligament and tendon complex attached to the arcuate fragment using a Krackow suture configuration and securely tied. Finally, the first suture tape is passed through the surrounding soft tissues and tied to reinforce fixation and enhance construct stability (Figure 2 – schematic and Figure 5).

**Figure 5:**
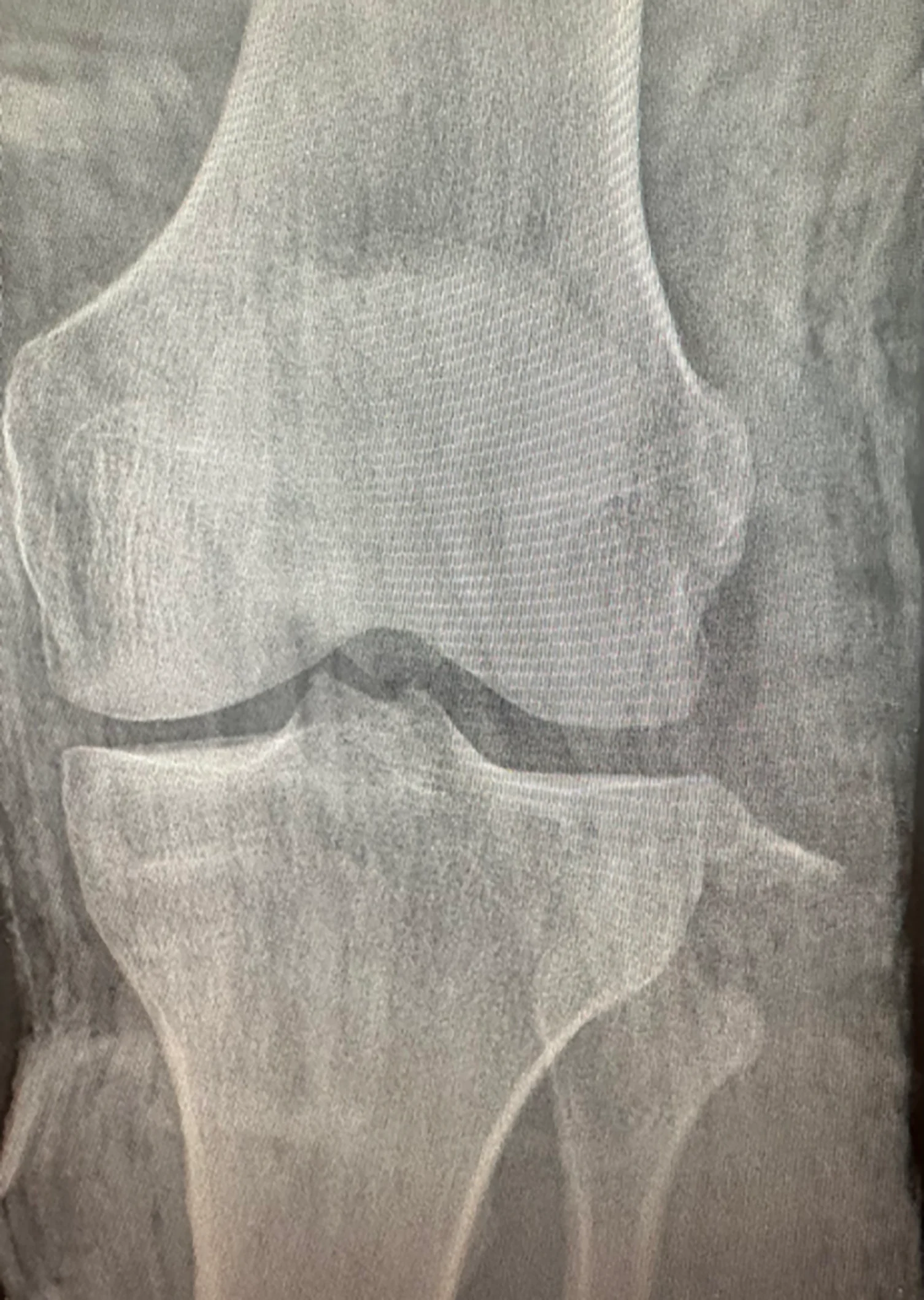
Pre-op X-Ray Radiographic evaluation demonstrated the fibular head avulsion.

The tightening of the sutures and the fixation are done in 300 degrees of flexion. The nerve is then released, and the wound is closed.

Postoperative regimen: The patient wears a knee splint or brace for 6 weeks in full extension, non-weightbearing; 6–8 weeks partial weight-bearing and active knee range motion, and 8–12 weeks full range of motion and full weight-bearing [8] (Figures 6 and 7).

**Figure 6:**
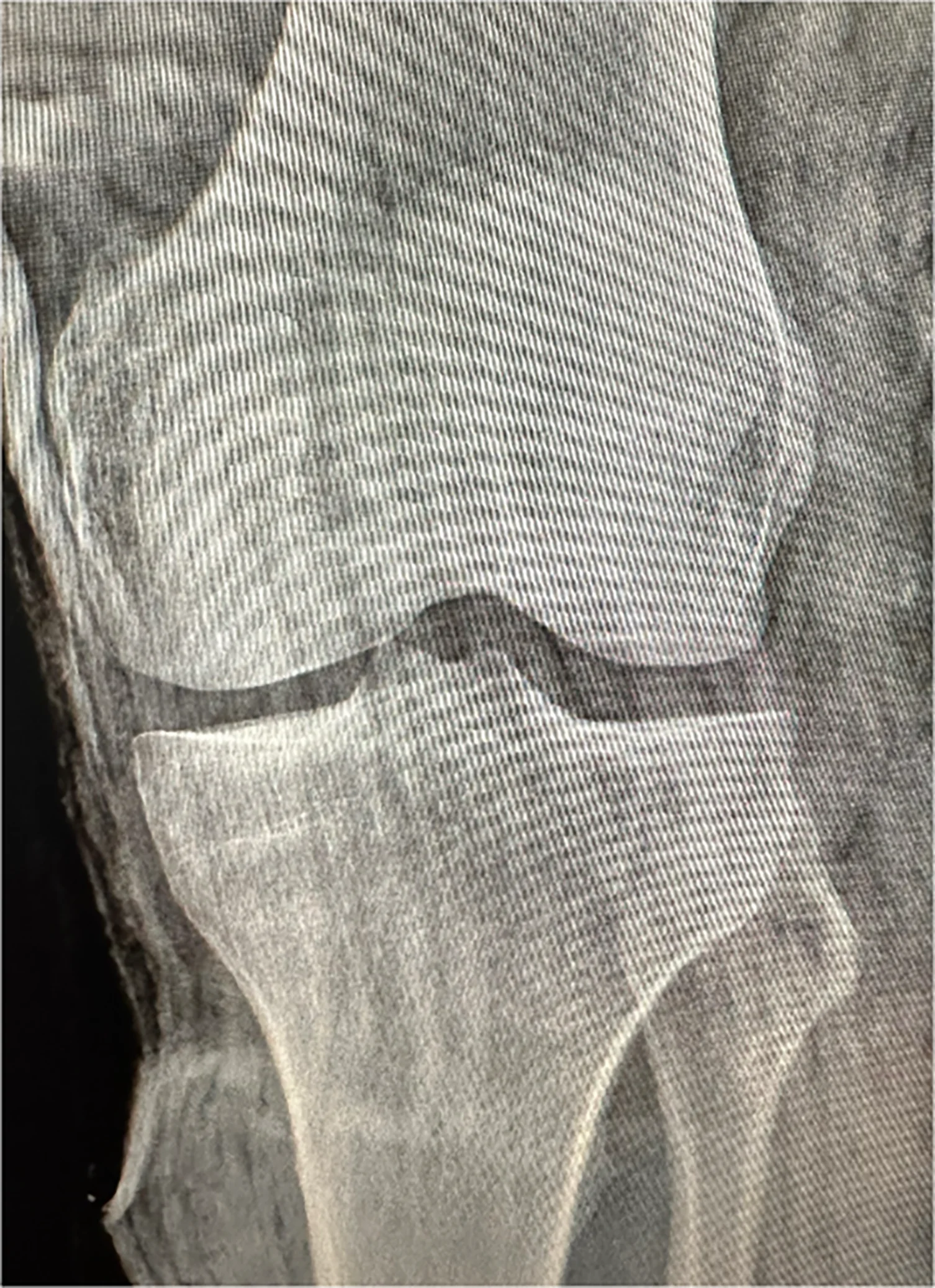
Day 1 Post-Op, immediate postoperative imaging confirming anatomic reduction and stable fixation.

**Figure 7:**
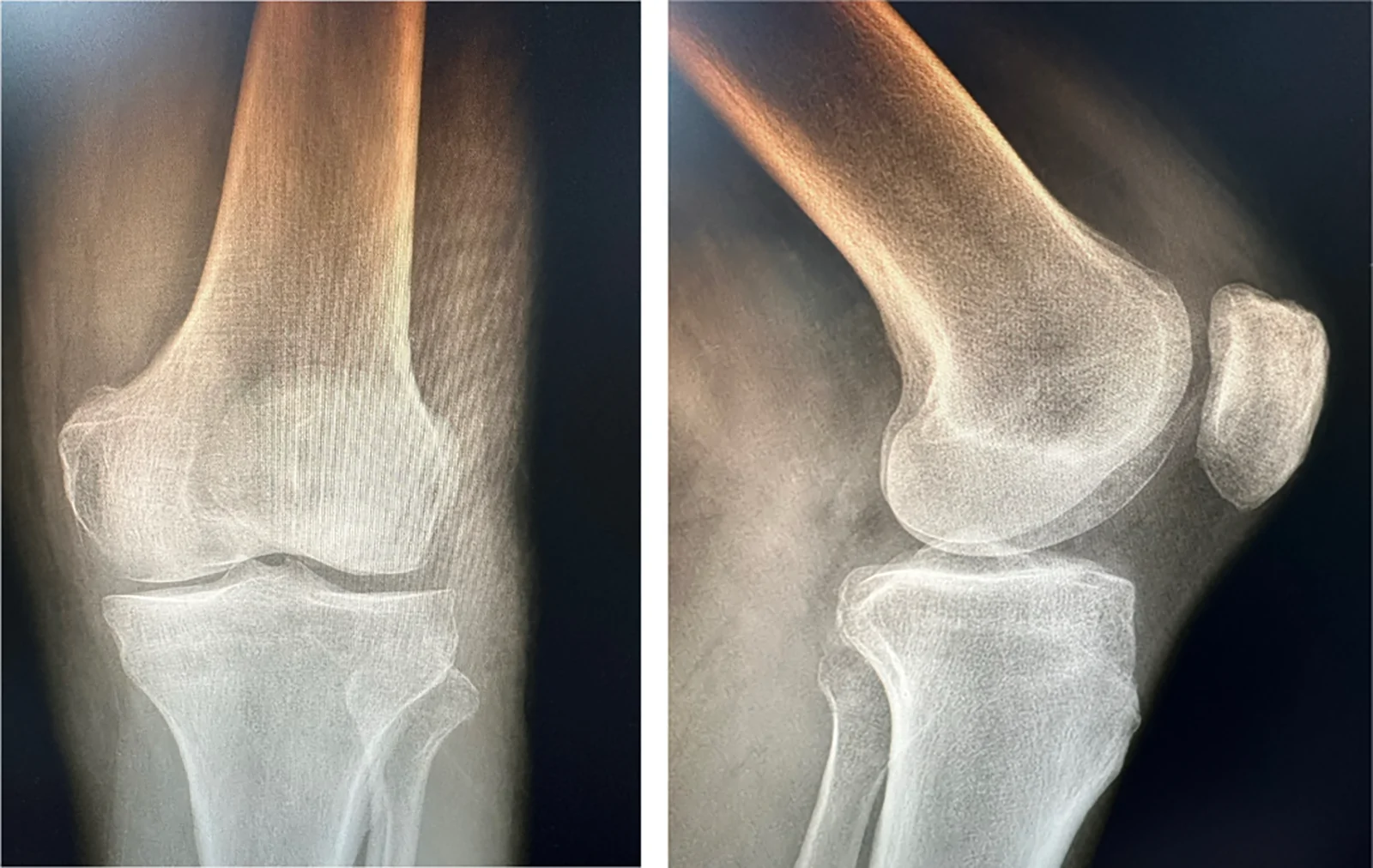
Three months Post-Op, revealing maintained alignment with evidence of fracture union.

## Discussion

Arcuate fractures of the proximal fibula are rare injuries. In a retrospective review of 2,318 knee injuries, only 13 cases involved arcuate fractures, representing an incidence of approximately 0.6% [1]. Despite their low frequency, these fractures are clinically significant because they often involve the posterolateral corner (PLC), a critical stabilizer of the knee.

The primary goal of surgical management of arcuate fractures is to achieve stable fixation of the avulsed fragment while restoring the anatomical and functional integrity of the PLC. The technique described in this study offers satisfactory results. It provides secure fixation, has a relatively short learning curve, and results in a low-profile construct that minimizes implant prominence and soft tissue irritation. However, a potential limitation is the risk of unequal suture tension during knot tying, which may compromise anatomic reduction and potentially contribute to delayed union or nonunion.

Cohen et al. retrospectively analyzed 48 proximal fibular fractures and proposed a classification system based on fragment size and anatomical involvement [3]. Pattern 1 consisted of small avulsion fractures of the fibular styloid; Pattern 2 involved avulsion of the cortical rim of the upper fibular head, corresponding to classic arcuate fractures of the arcuate complex; and Pattern 3 included fractures extending into the metaphyseal region of the fibular head. In their cohort, 22.9% of fractures were classified as Pattern 1, 12.5% as Pattern 2, and the majority (64.6%) as Pattern 3, underscoring the variability in fracture morphology and the need for individualized treatment strategies.

Management of arcuate fractures depends largely on fragment size, displacement, and associated ligamentous injuries. Spadafore and Khodaee reported successful nonoperative treatment of an isolated proximal fibular avulsion fracture in a young woman following low-energy trauma, with no associated ligamentous disruption [9]. Conservative management with rest, nonsteroidal anti-inflammatory drugs, and a structured rehabilitation program resulted in complete symptom resolution and return to baseline function within 10 weeks, suggesting that nonoperative treatment may be appropriate in select cases without PLC instability.

Several surgical techniques have been described for unstable or displaced arcuate fractures. Srimongkolpitak et al. detailed an open technique performed with the patient supine and the knee flexed to 90° [8]. Through a lateral approach, the fibular styloid fragment was exposed while carefully protecting the common peroneal nerve. The biceps femoris tendon, lateral collateral ligament (LCL), and popliteofibular ligament (PFL) were individually sutured using nonabsorbable sutures. Following anatomic reduction and temporary K-wire fixation, a tibiofibular tunnel was drilled, and the sutures were secured with a suspensory fixation device, stabilizing both the fracture and the proximal tibiofibular joint.

Biomechanical considerations also play a role in technique selection. Vojdani et al., in a cadaveric study, demonstrated that fixation using a spiked washer combined with an intramedullary screw provided greater load-to-failure strength compared with suture-based repairs [10]. Nevertheless, further investigations are required to assess cyclic loading behavior and the ability of this construct to restore native knee biomechanics.

De Alencar Neto et al. reported a rare case of bilateral fibular head avulsion fractures associated with Segond fractures [11]. In their case, one side demonstrated a comminuted fibular head fracture with an intact conjoint tendon, while the contralateral side exhibited multiple microfragments. Both sides were treated with transosseous fixation using Krackow sutures to secure the PLC structures to the fibular head, achieving satisfactory stabilization [12].

Minimally invasive approaches have also been explored. Zhang et al. described an all-arthroscopic technique utilizing a suture anchor for fixation of arcuate avulsion fractures [13]. Through posterolateral, posteromedial, and trans-septal portals, the avulsed fragment and PFL were visualized and secured. This technique provided stable fixation while minimizing soft tissue disruption and demonstrated promising early clinical outcomes.

More recently, a novel technique employing an adjustable-loop cortical suspension device during PLC reconstruction has been introduced [14]. In a retrospective series of nine patients, most achieved satisfactory clinical outcomes with no residual instability at final follow-up. Radiographic evaluation at six months confirmed fracture union in the majority of cases, with only one patient demonstrating incomplete union despite maintained reduction. These findings suggest that adjustable-loop cortical suspension devices represent a reliable alternative for stabilizing arcuate fractures, particularly in the setting of concomitant PLC reconstruction.

## Conclusion

The surgical technique described provides stable fixation while effectively restoring PLC integrity, offering a great alternative to current existing techniques with the advantages of a low-profile construct, decreased initial costs, and reproducibility. Although the risk of unequal suture tension represents a potential limitation, careful surgical technique can mitigate this concern. Overall, this method represents a reliable and effective option for managing unstable arcuate fractures, particularly in the setting of PLC involvement. Further comparative and biomechanical studies with larger patient cohorts are warranted to validate long-term outcomes and to establish the optimal fixation strategy for these uncommon injuries.

## Data Availability

All data supporting the findings of this study are included within the article. Further details are available from the corresponding author upon reasonable request.
